# Cost-effectiveness analysis of artificial intelligence-based diabetic retinopathy screening in rural China based on the Markov model

**DOI:** 10.1371/journal.pone.0291390

**Published:** 2023-11-16

**Authors:** Huilin Li, Guanyan Li, Na Li, Changyan Liu, Ziyou Yuan, Qingyue Gao, Shaofeng Hao, Shengfu Fan, Jianzhou Yang

**Affiliations:** 1 Department of Ophthalmology, Heji Hospital Affiliated to Changzhi Medical College, Changzhi, 046000, China; 2 Postgraduate Department, Changzhi Medical College, Changzhi, 046000, China; 3 Shenzhen Longgang Otorhinolaryngology Hospital, Shenzhen, 518100, China; 4 Department of Foreign Languages, Changzhi Medical College, Changzhi, 046000, China; 5 Department of Public Health and Preventive Medicine, Changzhi Medical College, Changzhi, 046000, China; Cairo University Kasr Alainy Faculty of Medicine, EGYPT

## Abstract

This study assessed the cost-effectiveness of different diabetic retinopathy (DR) screening strategies in rural regions in China by using a Markov model to make health economic evaluations. In this study, we determined the structure of a Markov model according to the research objectives, which required parameters collected through field investigation and literature retrieval. After perfecting the model with parameters and assumptions, we developed a Markov decision analytic model according to the natural history of DR in TreeAge Pro 2011. For this model, we performed Markov cohort and cost-effectiveness analyses to simulate the probabilistic distributions of different developments in DR and the cumulative cost-effectiveness of artificial intelligence (AI)-based screening and ophthalmologist screening for DR in the rural population with diabetes mellitus (DM) in China. Additionally, a model-based health economic evaluation was performed by using quality-adjusted life years (QALYs) and incremental cost-effectiveness ratios. Last, one-way and probabilistic sensitivity analyses were performed to assess the stability of the results. From the perspective of the health system, compared with no screening, AI-based screening cost more (the incremental cost was 37,257.76 RMB (approximately 5,211.31 US dollars)), but the effect was better (the incremental utility was 0.33). Compared with AI-based screening, the cost of ophthalmologist screening was higher (the incremental cost was 14,886.76 RMB (approximately 2,070.19 US dollars)), and the effect was worse (the incremental utility was -0.31). Compared with no screening, the incremental cost-effectiveness ratio (ICER) of AI-based DR screening was 112,146.99 RMB (15,595.47 US dollars)/QALY, which was less than the threshold for the ICER (< 3 times the per capita gross domestic product (GDP), 217,341.00 RMB (30,224.03 US dollars)). Therefore, AI-based screening was cost-effective, which meant that the increased cost for each additional quality-adjusted life year was merited. Compared with no screening and ophthalmologist screening for DR, AI-based screening was the most cost-effective, which not only saved costs but also improved the quality of life of diabetes patients. Popularizing AI-based DR screening strategies in rural areas would be economically effective and feasible and can provide a scientific basis for the further formulation of early screening programs for diabetic retinopathy.

## Introduction

Diabetic retinopathy (DR) is a common chronic complication of diabetes mellitus (DM). In the population with DM, the global prevalence of DR is approximately 35% [1] and 23% in China [2]. An upward secular trend is observed in the prevalence of DR as a result of low levels of awareness, insufficient education, neglect and unsatisfactory therapeutic effects for DR among patients with DM, which results in DR being one of the main causes of global blindness for adults of working age [3–6]. In 2015, the global number of people with blindness and moderate to severe visual impairment due to DR was 4 million and 26 million, respectively [6]. The number of DR patients is projected to increase to 191 million by 2030 [7]. Since the existence and progression of DR are associated with substantial increases in health costs [5,8], DR may become a great economic burden for the health care system in the future.

DR screening has been proven to be cost-effective by reducing vision loss [9–12]. Traditional DR screening was performed manually by an ophthalmologist or primary care practitioner using direct and indirect ophthalmoscopy and fundus photos for DR classification, which is time consuming and stressful for medical staff. Meanwhile, the disease burden of DR varies between urban and rural areas. For example, in China, the prevalence of DR among adults with type 2 diabetes is higher in rural areas (29.1%) than in urban areas (18.1%) [2]. In addition, while approximately 84.49% to 90.32% of rural patients with DM receive medical services in hospitals below the county level [13], there are only 0.05 and 0.02 ophthalmologists per 10,000 population in medical institutions below the county level in Central and Western China, respectively, which means that medical resources for ophthalmology are relatively scarce [14]. At the same time, due to the different distances from rural to urban areas, the DR screening rate of patients in rural areas may improve compared with urban hospital screening rates but will still be lower than 100% [15]. Regular screening by urban ophthalmologists in the countryside is not a long-term solution. Therefore, regular screening for DR in rural populations with DM undoubtedly faces great challenges.

Artificial intelligence (AI) automatic grading using a deep learning system has great potential in DR screening. With the development of AI, the DR screening mode gradually developed from an initial manual classification to automatic classification, from opportunistic and systematic screening to teleophthalmology-based DR screening, and even to using smartphone-based fundus photography for screening. The average reading time for AI can be as short as 1.62±0.67 s, which is equivalent to the time of experienced ophthalmologists with senior titles [16]. In resource-poor areas, AI-based screening has good consistency with ophthalmologist screening for DR, with an accuracy of 97–99% [17]. In addition, AI can provide an immediate diagnosis of DR, so AI-based screening can actively alleviate the pressure on ophthalmology medical resources and ophthalmologist shortages. In addition, AI-based screening not only improves the screening compliance and satisfaction of rural patients with DM but also saves them time, transportation costs and their vision while they wait to see a doctor, which thus also reduces the economic burden of the disease on the health system [18–21]. The change in DR screening strategy by AI is expected to bring new breakthroughs in the prevention, diagnosis and treatment of DR in DM patients and improve the cost-effectiveness of screening [22]. However, before these new technologies can be implemented, evidence is needed to demonstrate their clinical effectiveness and cost-effectiveness. Therefore, many countries and regions have conducted a variety of model-based health economic analyses to assess the long-term cost-effectiveness of different DR screening strategies, including the Markov model, which is more suitable for the cost-effectiveness study of chronic diseases with a long history and high risk of recurrence [23–37]. However, relevant research data about the cost-effectiveness of AI-based DR screening in China are limited, especially in rural areas. At present, in the research report published by Huang et al. in 2022, the Markov model was first used to conduct an economic evaluation of the cost-effectiveness of AI-based DR screening in rural China [37]. In 2020, a research project of a DR screening model based on an AI diagnosis system (Clinical Registration No.: CHICTR 200003283) was carried out in rural areas of Changzhi city, Shanxi Province. Our study used this project as the supporting project to analyze the cost-effectiveness of AI-based screening for DR based on a Markov model in rural areas of China to conduct health economic evaluations that will provide a basis for the implementation of early DR screening programs in rural areas.

## Materials and methods

### Investigation sites

Changzhi city, Shanxi Province is a typical city in Central and Western China located at the top of Taihang Mountain, with a resident population of 3,151,700 (by 2021) and a gross domestic product (GDP) of 231.11 RMB (approximately 32.33 billion US dollars), ranking 133rd among all cities in China. Changzhi city has jurisdiction over 4 districts and 8 counties, of which 1,384,745 people live in rural areas, accounting for 43.53% of the permanent population in the city. According to different economic levels, this study randomly selected Shangdang District (Changzhi County, which governs 6 towns and 5 townships, with a GDP of 21.64 billion RMB (approximately 3.01 billion US dollars)), Lucheng District (Lucheng County, which governs 4 towns and 3 townships, with a GDP of 12.54 billion RMB (equivalent to 1.74 billion US dollars)) and Huguan County (which governs 5 towns and 7 townships, with a GDP of 125.4 billion RMB (approximately 17.44 billion US dollars)) for DR screening, which represent the high, medium and low economic levels of Changzhi city, respectively.

### Ethics statement

The procedures of this study were in accordance with the Declaration of Helsinki and were approved by the Ethics Committee of Heji Hospital affiliated with Changzhi Medical College (approval no.: 2019016). Written informed consent was obtained from all participants.

### DR screening

#### Questionnaire design

The Ophthalmology Department of Heji Hospital affiliated with Changzhi Medical College set up a program implementation team that was responsible for providing screening equipment and monitoring the quality of screening, arranging the screening process, designing the questionnaire, and training the medical staff in township hospitals to set up a screening team.

#### Patient recruitment

The participants of this study included individuals with type 1 and type 2 DM, aged 18 years and above, who had registered in the DM management information database of the Changzhi National Basic Public Health Information System in Shanxi Province (by July 1, 2020). In this database, DM patients with accurate basic information and compliance with community management were recruited as screening participants, the list of whom was distributed to township health administrators, who verified and confirmed the DM patients on the list and established a free screening list. Within one month before the start of the screening, we organized the township staff to put up posters, conduct telephone and door-to-door calls according to the list, clearly explain the purpose, importance, content, benefits and possible hazards of this screening, and suggest that the patients attend the DR screening in township hospitals at the appointed time. At the screening site, the staff clearly informed the patients again about the purpose, importance, content, benefits and possible hazards of this screening and asked them to sign informed consent forms. In addition, all eligible and consenting patients filled out a baseline questionnaire on site to collect information about sociodemographic factors, ophthalmology-related history, general health status and ophthalmology-related diagnoses and treatment.

#### Site examination

All participants underwent the following examinations.

1. Vision examination: In a well-lit room, our staff measured the uncorrected/corrected distance visual acuity of each eye of the participants with a logMAR chart (Brien Holden Institute of Vision, Australia);
2. Slit-lamp microscopic examination: The slit-lamp microscopic examination of the anterior segment of the eye was performed by an experienced ophthalmologist to identify anterior segment diseases that affect vision;
3. Fundus image acquisition: In a relatively dark room, two fundus images of the undilated pupil were taken at 45° by a professionally trained eye nurse using a Zeiss VISUCA500 fundus camera (Carl Zeiss, Jena, Germany) for each eye of the patient (with the macula and optic disc as the centers).

#### Exclusion criteria

Participants who met any of the following criteria were excluded from further consideration: (1) inability to cooperate with the examination due to serious physical and/or other diseases; (2) refractive media opacity, such as corneal ulcers, corneal leukoplakia, severe cataracts, vitreous hemorrhage and massive exudation, that precluded a detailed fundus examination; and (3) poor quality of the acquired image.

#### DR grading and follow-up

DR was graded according to the International DR Severity Grading Scale [38]: (1) no DR (no abnormal change); (2) mild nonproliferative diabetic retinopathy (NPDR) (only microaneurysm); (3) moderate NPDR (microaneurysms, milder than severe NPDR); (4) severe NPDR (without proliferative diabetic retinopathy (PDR) manifestations, including any of the following: more than 20 retinal hemorrhages in any quadrant; venous beading changes in > 2 quadrants; prominent intraretinal microvascular abnormalities in > 1 quadrant); and (5) PDR (any of the following changes: neovascularization, vitreous hemorrhage, or subretinal hemorrhage).

The final result of the diagnostic classification was the result of severely diseased eyes. As clinically significant cystoid macular edema could not be confirmed by fundus images, the diagnosis was not mentioned in this study.

#### DR screening mode

Fundus images were collected for screening in two modes:

1. AI-based screening mode: The AI diagnostic system used in this study was EyeWisdom, AI analysis software from Zhiyuan Huitu for fundus images (Visionary Intelligence Ltd., Beijing, China), which was based on 25297 fundus image databases (21512 from the Kaggle database and 3785 from Henan Eye Hospital and Beijing Union Medical College Hospital). To use this software, more than one million manifestations of retinopathy were manually marked by fundus disease experts to develop the deep learning system and to train the network data by using the You only look once (YOLO) detection system [39]. EyeWisdom is an online retinal image analysis platform. In this study, after the retinal images were uploaded, the AI diagnostic system automatically graded and generated a single-page graded report including referral recommendations for patients [40]. As the image quality and diagnosis calibration were confirmed, the staff would print the examination report and carry out patient health education or guide them to the designated hospital for treatment according to the recommendations of their report results.
2. Ophthalmologist screening mode: The fundus images were transmitted to two experienced ophthalmologists from high-level hospitals in the urban area to grade. If their grading results were inconsistent, these fundus images were sent to a chief physician to read and make a final grading result. All the grading results and follow-up suggestions of the ophthalmologists were returned to the patients within 1 week.

Eye examinations at the screening site were provided free of charge to the patient, whereas the patient was responsible for his own examination and treatment at the hospital.

#### Follow-up

Patients without DR or with mild NPDR were followed up once a year, patients with moderate NPDR were followed up every 6 months and those with severe NPDR or PDR were referred to the Heji Hospital affiliated with Changzhi Medical College for further evaluation and treatment. Subsequent follow-up examinations included visual acuity, intraocular pressure measurement, slit-lamp microscopic examination, fundus photography, and fundus fluorescein angiography for patients with NPDR or PDR. Laser photocoagulation and vitrectomy were provided as appropriate.

### Project quality control

1. The Ophthalmology Department of Heji Hospital affiliated with Changzhi Medical College set up a scheme implementation team, which was responsible for the training of medical staff and the monitoring of screening quality, to ensure that a unified scheme could be implemented in different towns and villages at different times.
2. The investigators were trained on the informed consent form and questionnaire by the project leader, with emphasis on confusing or unclear issues, drawing up a unified standard scope, strengthening questioning skills and mitigating information bias caused by investigators.
3. Before the formal screening, the design of the questionnaire, the collection of on-site data, the calibration of diagnostic results and the data input were strictly controlled to ensure the feasibility and effectiveness of the screening.
4. The database was added to Excel to compare and check many times to find errors and omissions to improve the authenticity of the data.

### Data collection

1. Through DR screening, we collected data from both AI-based screening and ophthalmologist screening, which included the results of DR grading and questionnaires and the transportation costs for patients to participate in the screening.
2. By consulting the project manager, the costs related to the implementation of the screening project, including equipment, manpower, mobilization and recruitment, training, transportation and other project-related costs, were also collected.
3. The costs of follow-up examinations, laser treatment, and vitrectomy for DR patients were collected from Heji Hospital affiliated with Changzhi Medical College.

### Markov model-based assessment

#### Markov model principles

The Markov model is a process of simulating random events with time series, which is characterized in that the state of a certain time point in the sequence is only related to the current value but not to the value at any time in the past. Such a special property is called "no aftereffect". Therefore, the Markov model, as one of the commonly used mature decision analysis methods, is especially suitable for the study of chronic diseases [41,42].

The application principle of Markov model decision analysis is that according to the research purpose and the natural history of the disease, the disease is divided into a number of different health states (i.e., Markov states). Within a certain period, each state transfers to another according to the transition probability to simulate the natural development process of the disease. Combined with the health effects and costs of each state, the long-term development as well as the effects and medical costs of the disease with different health interventions are estimated after multiple cycles.

**Fig 1:**
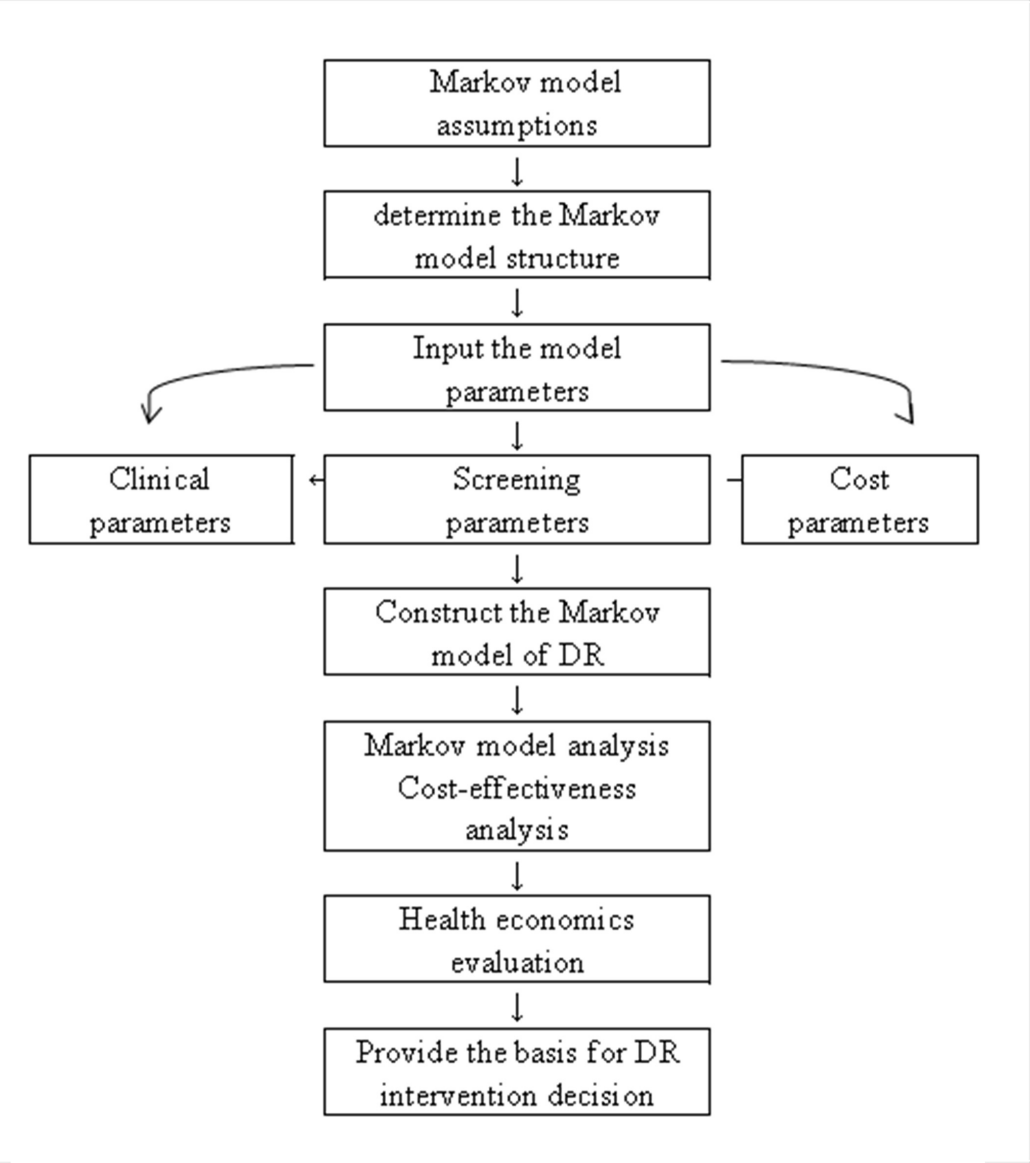
Flow chart of the Markov-based assessment in this study.

The flowchart of the assessment in this study is shown in Fig 1.

#### Markov model assumptions

In this study, a Markov model was used to simulate the development of DR in the next 50 years in a hypothetical cohort of 10,000 rural patients with DM. To simplify the calculation, the following assumptions were made for the model:

1. All patients with DM did not have diabetic macular edema.
2. Compliance for screening, follow-up and treatment of patients with type 2 DM was 100%.
3. The DM patients in the nonscreening group did not receive any intervention, so DR developed naturally, whereas DM patients in the AI-based screening and ophthalmologist screening groups received laser intervention according to the treatment guidelines for DR.
4. The model parameters of the nonscreening group and the screening group were the same, except for the difference in initial probabilities, transition probabilities and costs of severe NPDR and PDR.
5. The sensitivity and specificity of the AI-based screening group and the ophthalmologist screening group for the diagnosis of DR were consistent with the results of the study by He et al. [43] and Li et al. [44], respectively.
6. In each cycle, patients with DM could only transition to one state according to the transition probability, rather than to multiple states at the same time. In addition, patients with DR were not allowed to return to the fully healthy state, that is, the state without DR.
7. The costs for no DR, mild NPDR, diabetes, blindness, and both disease-related deaths in the model were all 0.

#### Determining the Markov model structure

The basic parameters of the Markov model include the Markov state, transition probability, cost, effect, cycle period, and cycle termination condition. In this study, one year was used as the cycle period, and quality-adjusted life years (QALYs) were used as the effect index.

**Fig 2:**
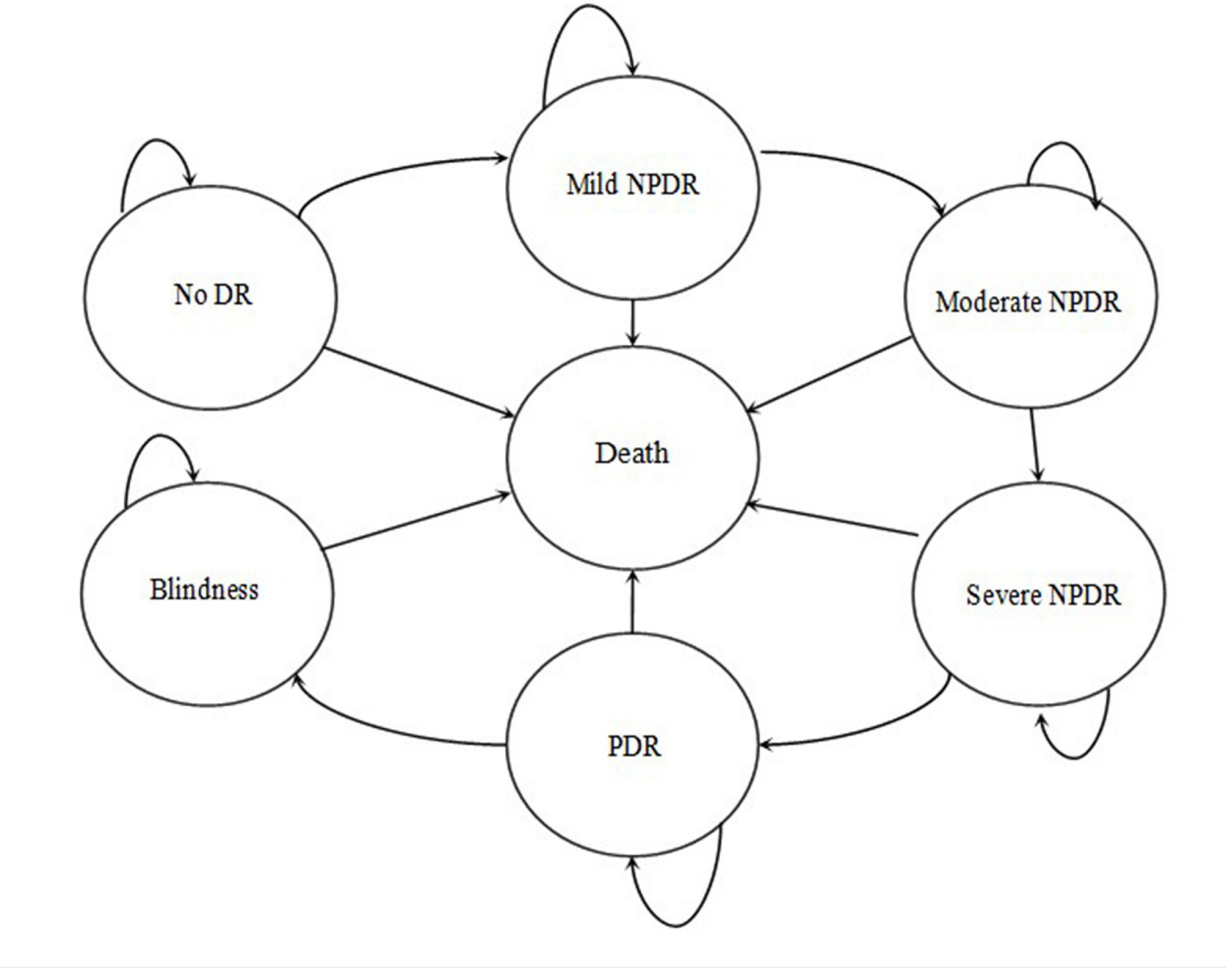
Markov model structure. DR = diabetic retinopathy; NPDR = nonproliferative diabetic retinopathy; PDR = proliferative diabetic retinopathy.

The Markov state diagram of DR was established according to the criteria of the Early Treatment of Diabetic Retinopathy Study (ETDRS). In the model, patients with diabetes were divided into seven Markov states: no DR, mild NPDR, moderate NPDR, severe NPDR, PDR, blindness, and death. Assuming that the patient’s initial state was one of the seven states, the transit paths between all states in each cycle were as follows: (1) patients without DR might maintain their original state or progress to mild NPDR; (2) mild NPDR might remain unchanged or progress to moderate NPDR; (3) moderate NPDR might remain unchanged or progress to severe NPDR; (4) severe NPDR might remain unchanged or progress to PDR; (5) PDR might remain unchanged or progress to blindness; and (6) in any state, the patient was at risk of death. The probability that one state transitions to another state within a unit cycle is called the transition probability. The Markov model structure is shown in Fig 2.

We compared three different screening strategies for DR: no screening, AI-based screening and ophthalmologist screening. It was assumed that the individuals in the model were individually enrolled into one of three screening groups: the nonscreening group (natural history), the AI-based screening group and the ophthalmologist screening group. DR was in a state of natural development without any intervention for participants in the nonscreening group. In the AI-based screening group and the ophthalmologist screening group, the population with DM was screened for DR by using fundus photography. DR-positive and DR-negative patients who did not need referrals were followed up according to the follow-up guidelines for DR, while DR-positive patients requiring further investigation and treatment were referred to an ophthalmology clinic. Assuming that patients diagnosed with severe NPDR and PDR would be treated with laser therapy, the risk of progression of DR after treatment would be reduced, but the patient would not return to their previous health state.

#### Determination of model parameters

Through PubMed, GeenMedical, the China National Knowledge Infrastructure (CNKI), Wanfang Database, etc., the relevant literature and research were retrieved with "Markov, diabetic retinopathy, screening, cost-effectiveness and economic evaluation" as the keywords to obtain the DR prevalence, transition probability, health utility value, cost, mortality, and other parameters required in the process of constructing the Markov model. Other parameters were derived from the raw data of this study.

1. Clinical parameters. The clinical parameters required for the model in this study included the prevalence and annual transition probability of DR, relative risk (RR) of laser treatment, natural mortality of the rural population in China and mortality multiplier for diabetes and blindness. The prevalence of DR was estimated based on the DR epidemiological data of rural populations with DM in Shanxi Province reported by Wang et al. [45]. The annual transition probability was derived from a study by Tung et al. [46]. The relative risk of laser therapy was derived from Evans JR et al., who reported a value of 0.49 [47]. The natural mortality rate of the rural population in China was 7.07% [48] according to 2019 data reported in the China Health Statistics Yearbook 2020. The mortality multiplier for diabetes and blindness was estimated by Zhang et al. [49].
2. Screening parameters. The screening parameters included the positive detection rate of DR screening derived from the original data of the supporting project, as well as the sensitivity and specificity of AI-based screening and ophthalmologist screening derived from the studies by He et al. [43] and Li et al. [44].
3. Health utility value. With the change in new medical models, modern medical efficacy not only takes survival time as an evaluation index but also attaches more importance to the evaluation of quality of life [50]. The occurrence and development of different diseases and various clinical screening, diagnosis and treatment interventions will have a certain impact on the quality of life and survival time of patients. QALYs are indicators representing the quality of life status of people, which have taken into account the effects of interventions such as clinical screening, diagnosis and treatment on the prolongation of life and improvement of quality of life of the population at the same time.
  The health utility value is a quality of life adjustment weight used to calculate QALYs, which can convert the quality of life years of patients with diseases or disabilities into the equivalent of those of fully healthy people, reflecting individual preferences for health status. QALYs of the patient within a certain cycle time = the health utility value of the state under the cycle × cycle time [42]. The health utility value data in this study were all from the study by Tung et al. [51].
4. Costs. In the view of the health system, costs only included direct costs (direct medical costs and direct nonmedical costs). In this study, the direct medical costs included the costs of screening, follow-up examinations and treatment. Specific costs were as follows:
  a. Costs of screening. In this study, the costs of screening of a project were calculated by estimating and summarizing the costs of individual work items or resource consumables based on the project site. Since the screening program of the supporting project of this study was centralized screening for the diabetic population instead of routine screening (that is, 250 working days per year, except for screening conducted on 11 legal holidays and 104 rest days), the coverage of the screened population was still not wide enough. Therefore, we estimated the average annual number of people screened during routine screening; according to the screening sites in the supporting project, the average daily number of people screened was 65, which would be reduced by switching to routine screening. It was estimated that the daily screening workload was 50 people, so 12,500 people could be screened annually. Finally, the per capita cost of screening was estimated according to the average annual costs of screening and the average annual number of people screened. Screening costs included direct screening costs and indirect screening costs.

The direct screening costs for this study included the following: (1) Equipment costs, including a fundus camera with a cost of 196,000 RMB (approximately 27,256.29 US dollars) and a service life of 8 years; AI software with a cost of 96,000 RMB (approximately 13,350.02 US dollars) and a service life of 50 years, which is equivalent to the copyright protection period; and (2) manpower costs, including the service fees of screening personnel and ophthalmologists. As the research relied on the project in which ophthalmologists from high-level hospitals go to the countryside to conduct DR screening, based on their average daily salary, the service fee of ophthalmologists was 300 RMB (41.73 US dollars)/day, and the service fee of screening personnel was 600 RMB (84.43 US dollars)/day. For the AI-based screening group, the annual direct costs of screening were 176,420 RMB (approximately 24,538.56 US dollars), with the equipment costs that included the AI software cost, fundus camera cost and manpower costs that included the service fees of the screening personnel. For the ophthalmologist screening group, the average annual direct costs of screening were 249,500 RMB (approximately 34,703.39 US dollars), including the equipment costs that included the fundus camera cost and the manpower costs that were composed of the service fees of the screening personnel and ophthalmologists. The direct costs of screening are shown in Table 1.

**Table 1:** The direct costs of screening (the cost was calculated based on a Chinese yuan: US dollar ratio of 7.189: 1).

Cost items	Cost (US dollars)	Duration of use (years)	Frequency	Annual cost (US dollars)
Equipment				
Fundus camera	27,261.98	8	1	3407.75
AI software	13,352.81	50	1	267.06
Manpower				
Screening personnel	83.46		250	20,863.76
Ophthalmologist	41.73		250	10,431.88
Total				34,970.44

Indirect costs of screening referred to the costs of screening projects, including the recruitment costs of screening mobilization, transportation costs of staff and equipment, and equipment maintenance costs. The screening mobilization and recruitment costs included the production of publicity posters and banners, the printing of health education manuals and questionnaires, the training of screening personnel and township medical personnel, and the cost of rural drivers.

The costs in this study were estimated from the related costs of the project in one year: 50 publicity posters with a unit price of 20 RMB (2.78 US dollars) were printed in each township for a total cost of 23,000 RMB (3,199.11 US dollars) for 23 towns. One copy of the publicity banner was printed for recycling at the price of 15 RMB (2.09 US dollars).

The annual requirements of the health education manual and questionnaire were 12,500 copies each, the total costs of which were 10,875 RMB (1,512.62 US dollars) with a unit price of 0.6 RMB (0.08 US dollars) for the former and 0.27 RMB (0.04 US dollars) for the latter. With a training duration of one hour at the price of 600 RMB (83.46 US dollars) every time, a total of 23 trainings were conducted for screeners and township doctors, which cost 13,800 RMB (1,919.47 US dollars). The time costs of township doctors were estimated by estimating the average salary, which was based on the monthly salary income of 3,500 RMB (486.82 US dollars) and the monthly salary days of 21 days. The unit hourly salary was equal to the monthly salary income divided by the multiplier of monthly salary days and 8 hours, so that the total training time costs for 102 people participating in training were 2,142 RMB (297.93 US dollars) at a unit time cost of 21 RMB (2.92 US dollars) per person for township doctors. The annual total manpower costs of drivers going to the countryside were 50,000 RMB (6,954.59 US dollars), which were calculated by multiplying 20 RMB (2.78 US dollars)/day by 250 days. Due to the different distances between rural areas and cities, it took 23 trips for the equipment to be transported to 23 rural areas, so the estimated total distance for equipment transportation was 846 km by multiplying a single journey by the frequency between each location. As the single journey for staff was 114 km/day, the annual journey for transporting staff was 28,500 km. Therefore, the total journey for transporting staff and equipment every year was 29,346 km. Combined with the average transportation cost of approximately 6.40 RMB (0.89 US dollars)/liter and approximately 6.6 liters/100 km, that is, 0.4224 RMB (0.06 US dollars)/km, the total annual transportation costs were approximately 12,395.5 RMB (1,724.11 US dollars). The maintenance cost of the fundus camera was 2,000 RMB (278.18 US dollars) monthly, while the annual equipment maintenance costs were 24,000 RMB (3,338.20 US dollars). In summary, the estimated average annual indirect costs of screening were 136,227.75 RMB (18,948.15 US dollars).

According to the sum of the average annual direct costs and indirect costs of screening divided by the average annual screened population, the per capita cost of screening for the AI-based screening group and the ophthalmologist screening group was 25.01 RMB (3.48 US dollars) and 30.86 RMB (4.29 US dollars), respectively. The indirect costs of screening are shown in Table 2.

**Table 2:** Indirect costs of screening (the cost was calculated based on a Chinese yuan: US dollar ratio of 7.189: 1).

Items	Unit cost (US dollars)	Frequency	Annual cost (US dollars)
Recruitment			
Publicity posters	2.78	1150	3199.11
Promotional banner	2.09	1	2.09
Health education manual	0.08	12500	1043.19
Questionnaire	0.04	12500	469.43
Manpower cost of training	83.46	23	1919.47
Time cost of training	2.92	102	297.93
Manpower costs of driver	27.82	250	6954.59
Transportation costs	58.97	29346	1724.15
Equipment maintenance costs	278.18	12	3338.20
Total			18948.15

b. Follow-up costs. The follow-up costs referred to the costs of patients who were positive for DR screening and needed to be transferred to a higher-level hospital for further examination and treatment, including routine examination in an ophthalmology clinic, diagnosis and treatment by an ophthalmologist, two color fundus photos and fundus fluorescein angiography. According to the single cost of related examinations in Heji Hospital affiliated with Changzhi Medical College, the per capita diagnosis cost was 610.2 RMB (84.87 US dollars). The follow-up costs are shown in Table 3.

**Table 3:** The follow-up costs and treatment costs (the cost was calculated based on a Chinese yuan: US dollar ratio of 7.189: 1).

Items	Cost (US dollars)	Sensitivity analysis range
Follow-up costs		
Medical expenses	1.04	3.75–11.25
Routine ophthalmic examination	2.92	10.5–31.5
Fundus photo	24.48	88.0–264.0
Fundus fluorescein angiography	56.43	202.85–608.55
Treatment costs		
Laser photocoagulation	424.41	1525.65–4576.95
Vitrectomy	1647.01	5920.6–17761.8

c. Treatment costs. Treatment costs only referred to the medical expenses incurred during the treatment process of patients diagnosed with DR. Since most patients had visited different hospitals in succession, the detailed collection of these costs was not performed in this study. To simplify the model calculation, the surgical treatment costs of patients with DR were included for analysis, including laser photocoagulation and vitrectomy. The related costs were obtained from Heji Hospital affiliated with Changzhi Medical College to estimate the treatment costs of DR patients in different stages. The treatment costs are shown in Table 3.
d. Direct nonmedical costs. The direct nonmedical costs only referred to the transportation expenses incurred by the patient in the process of seeking medical treatment, mainly including the two-way transportation expenses for the patient to participate in DR screening and to visit a higher-level hospital. The diabetic patients in the supporting project of this study needed to go to the township health centers in their own villages or neighboring villages to participate in the screening. Through the on-site questionnaire, it was determined that the average transportation costs of each patient to and from the screening site and from a higher-level hospital were 2.74 RMB (0.38 US dollars) and 17.80 RMB (2.48 US dollars), respectively. Therefore, the per capita direct nonmedical cost was 20.54 RMB (2.86 US dollars). The direct nonmedical costs are shown in Table 4.

**Table 4:** Direct non-medical costs (the cost was calculated based on a Chinese yuan: US dollar ratio of 7.189: 1).

Items	Cost (US dollars)	Sensitivity analysis range
Per capita transportation cost		
Screening	0.38	1.35–4.05
Hospital visit	2.48	8.9–26.7
Total	2.86	10.27–30.81

(5) Discount rate. When the duration of the health intervention program or study treatment exceeds one year, the costs or outputs at different time points cannot be equivalently compared because the time value of money in different years is often different. To facilitate the comparison of costs or outputs at the same time point, it is necessary to convert the different future time points of cost and health utility values into the same time point according to a certain ratio. This process is called discounting. The converted ratio is the discount rate. According to the recommendation of the World Health Organization (WHO), a discount rate of 3% was used for both the cost and health outputs of this study [52].

#### Cost-effectiveness analysis

Cost-effectiveness analysis is the most commonly used economic evaluation method in the field of health economics at present. Relative effect indicators (such as morbidity and mortality) and absolute effect indicators (such as the number of people found and treated) are used as the measurement units of output or effectiveness instead of monetary units. The economic effects of different health intervention programs are compared and evaluated in terms of both cost and effectiveness to select the optimal program with the lowest unit effect cost, provide information for decision-makers and maximize the benefits of limited health resources.

There are three methods for cost-effectiveness analysis: (1) When the costs between the alternative solutions are basically the same, the solution with the largest effect is selected as the optimal solution; (2) When the effects of the alternative schemes are basically the same, the scheme with the lowest cost is selected as the preferred program; (3) When the cost and effect of alternatives change each other, the ratio of incremental cost to incremental effect, that is, the incremental cost-effect ratio, needs to be calculated. This method is currently in common use.

The results of the cost-effectiveness analysis are shown in the cost-effectiveness diagram. The evaluation indexes include the cost-effectiveness ratio (CER), which indicates the cost of unit effectiveness, and the incremental cost-effectiveness ratio (ICER), which indicates the cost of poor unit effectiveness between two alternatives. If we chose QALYs as an indicator of effect, the ICER indicates the cost of saving per QALY, which is used to measure the effectiveness of alternatives. Only the ICER was used as an indicator for analysis in this study. The specific calculation formula was as follows [53]:

ICER=△C/△E=(C1‐C2)/(E1‐E2),

where C and E represent the cost and effectiveness of the program, respectively.

Decision-makers are also limited by the impact of the budget while pursuing the maximum effect. When selecting strategies, it is generally necessary for them to consider the threshold of the ICER, or willingness-to-pay (WTP), which refers to the maximum additional cost that the decision-makers are willing to pay for the unit effect. The WTP threshold recommended by the WHO is used as the reference threshold, which is usually expressed as 1–3 times the per capita GDP. When the ICER is < the per capita GDP, the intervention plan is considered very cost-effective; when the ICER is < 3 times the per capita GDP, the intervention plan is considered cost-effective, that is, the increased costs of health interventions are worthwhile; and if the ICER is > 3 times the per capita GDP, the program is not considered cost-effective [54].

#### Sensitivity analysis

Sensitivity analysis is a process of testing the reliability of economic evaluation conclusions. In the cost-effectiveness analysis, there are many variable parameters that are uncertain, such as the discount rate, health effects, and changes in costs. Changes in any one of these parameters will lead to changes in costs or effects. Therefore, sensitivity analysis is required to test the reliability of the results or conclusions. Sensitivity analysis refers to changing model parameters within a certain range of estimates to evaluate the extent to which model parameters affect the results or conclusions [42].

Before performing the sensitivity analysis, it was necessary to set each parameter in the model to obey a certain distribution first and then to determine the probability value range of the model variables. In this study, the positive detection rate of DR screening, the prevalence rate of DR, the natural mortality rate of the rural population and the health utility value all had a beta distribution, the laser treatment effect had a logarithmic normal distribution and the cost had a gamma distribution. According to the guidelines of pharmacoeconomic evaluation in China and referring to other research practices [37,42,55], a range of ±25% was applied for the DR prevalence, positive detection rate and transition probability. The costs were in the range of ±50%. The minimum and maximum values were estimated from 95% confidence intervals for the health utility value, sensitivity and specificity of ophthalmologist and AI-based screening, relative risk of laser treatment, and mortality multiplier of diabetes and blindness, which were derived from the literature. According to the recommendation of the WHO, the discount rate ranged from 0%-6%.

A one-way sensitivity analysis was used to analyze all the uncertain parameters in the model, the results of which were represented by a tornado diagram that showed the varied range of the ICER when all the parameters varied singly and was convenient for us to select the parameters that had great impact on the model to analyze.

Probability sensitivity analysis was used to analyze the impact on results or conclusions when multiple parameters in the model were changed simultaneously.

It took 10,000 repeated samples across the ranges of the parameters by using Monte Carlo simulation technology to simulate and observe the influence of uncertain parameters on the model, where one value was randomly selected from the probability distribution of these parameters for operation each time. Monte Carlo simulation is also called the computer random simulation method and is a main method for studying distribution characteristics by setting a random process and repeatedly generating time series to calculate parameter estimators and statistics.

As the simulated queue population in the model individually passed through the model in turn, the current individual randomly entered different states of the next cycle according to the transition probability distribution among each state. Only when the last individual reached the absorption state could the next individual begin. According to the sample results obtained by simulating a large number of individuals, the cost, effect and ICER of different intervention measures were finally analyzed. This method was close to the actual situation, while all the possible results of the decision could be observed, and the degree of variation in the results could be estimated to make a better decision under uncertainty. The results could be displayed as a cost-effectiveness scatter plot, incremental cost scatter plot and cost-effectiveness acceptance curve (CEC), which represented the probability that the ICER of each alternative was lower than the WTP threshold under different WTP thresholds, that is, the probability of cost-effectiveness of the alternative proposal.

### Statistical analysis

Excel was used to establish a database to enter and complete the data. SPSS 22.0 software was used for data description and statistical analysis. The baseline characteristics of the cohort population used percentages to describe classified variables and means ± standard deviations to describe continuous variables. The diagnostic consistency between AI-based and ophthalmologist screening for DR was tested using the Kappa test. A Kappa value < 0.4 indicated that the two had poor consistency, a Kappa value < 0.75 indicated that the two had general consistency, and a Kappa value ≥0.75 indicated that the two had good consistency. The comparison between the two groups was performed using a two-tailed x^2^ test. The significant α value was 0.05, and the difference was considered statistically significant if P < 0.05.

The Markov decision model for DR was constructed using TreeAge Pro 2011 (TreeAge Software Inc., Williamstown, MA) software (S1–S3 Figs). After perfecting the required parameters of the model, a total of 50 cycles were run and simulated, and the cycle of the model was set as one year. Markov queue analysis and cost-effectiveness analysis were conducted by using a Markov model to simulate the long-term development of DR in a cohort of 10,000 patients with DM in rural China and the probability distribution of each state, the cost and effect of each cycle and the cumulative cost and effect under different DR screening strategies. Economic evaluation was conducted by comparing the ICER. In the queue population of the Markov model, the state transition was set to occur at the end of the cycle, which was actually a continuous process that could be changed at any time during the cycle, so semiperiodic correction was conducted on the model to make the state transition occur in the middle of the cycle on average. Finally, the sensitivity analysis of model parameters was carried out by the Markov model to observe the degree of influence of uncertain variables on the results or conclusions.

## Results

### Epidemiologic results of AI-based and ophthalmologist screening for DR

According to the DR screening data of the project, a total of 4488 DM patients with 8778 eyes participating in DR screening were included in this study, and the following data were excluded: 1) 284 eyes of 142 patients with nongraded binocular AI diagnosis; 2) 192 eyes of 97 patients with nongraded eyes; 3) incomplete information about 179 cases of vision deficiency/artificial eyes, sex in 744 cases, and age in 224 cases, resulting in a total of 2199 eyes. Finally, 6076 eyes (3057 right eyes and 3019 left eyes) of 3102 DM patients aged 27–87 years were analyzed, including 663 from Huguan County, 1514 from Lucheng County and 925 from Changzhi County. There were 1019 males and 2083 females, with an average age of 63.75 years (±8.424).

Tables 5–7 show the DR grading of the AI-based screening group and the ophthalmologist screening group. The positive detection rate of DR was 32.59% (1011 cases) in the AI-based screening group and 16.54% (513 cases) in the ophthalmologist screening group. Among them, 503 patients were diagnosed with DR, and 2381 patients were without DR in both the AI-based screening group and the ophthalmologist screening group. The diagnostic coincidence rate between the two groups was 92.97%, Kappa = 0.780, P = 0.014 (< 0.05), and the difference was statistically significant. The results showed that there was a high consistency between the AI-based screening group and the ophthalmologist screening group, and it was feasible for ophthalmologists to screen for DR instead of AI in rural areas.

**Table 5:** Comparison of DR screening results of 3102 DM patients between an ophthalmologist and AI.

	Ophthalmologist diagnosis	AI diagnosis system	Number of consistent diagnoses
DR	513	**843**	**503**
NDR	2589	**2259**	**2381**
Total	3102	**3102**	**--**

Notes: DR: Diabetic retinopathy; NDR: Non-diabetic retinopathy.

**Table 6:** Binocular DR grading of 513 DR patients screened by an ophthalmologist.

DR grading	Total eyes	Right eye	Left eye
NDR	177(17.25)	80(15.59)	97(18.91)
Mild NPDR	146(14.23)	78(15.20)	68(13.26)
Moderate NPDR	236(23.00)	123(23.98)	113(22.03)
Severe NPDR	386(37.62)	196(38.21)	190(37.04)
PDR	46(4.48)	27(5.26)	19(3.70)
No grading	35(3.41)	9(1.75)	26(5.07)
Total	1026(100.00)	513(100.00)	513(100.00)

Notes: DR: Diabetic retinopathy, NDR: Non-diabetic retinopathy, PDR: Proliferative diabetic retinopathy.

**Table 7:** Binocular DR grading of 1011 DR patients screened by AI.

DR grading	Total eyes	Right eyes	Left eyes
NDR	600(29.67)	239(23.64)	361(35.71)
Mild NPDR	700(34.62)	416(41.15)	284(28.09)
Moderate NPDR	482(23.84)	250(24.73)	232(22.95)
Severe NPDR	110(5.44)	52(5.14)	58(5.74)
PDR	92(4.55)	44(4.35)	48(4.75)
No grading	38(1.88)	10(0.99)	28(2.77)
Total	2022(100.00)	1011(100.00)	1011(100.00)

### Results of Markov model parameters

Before the cost-effectiveness analysis and sensitivity analysis of DR screening, the parameter inputs of the Markov model had to be improved. Table 8 shows the specific model parameter values and sensitivity analysis range. Other model parameters also included the DR positive detection rate and cost, among which the per capita cost of the AI-based screening group was 25.01 yuan (3.48 US dollars). The per capita cost of the ophthalmologist screening group was 30.86 yuan (4.29 US dollars), and the other specific cost parameters are shown in Tables 3 and 4.

**Table 8:** Markov model parameters.

Model parameters	Variable names in the model	Figures	Range of sensitivity analysis
The prevalence of DR in rural areas (%) [45]			
NDR	pNoDR	62.54	±25%
Mild NPDR	pMildNPDR	8.12	
Moderate NPDR	pModerateNPDR	14.49	
Severe NPDR	pSevereNPDR	10.22	
PDR	pPDR	4.63	
Transition probability [46]			
From NDR to mild NPDR	p_no_to_mild	0.07	±25%
From mild NPDR to moderate NPDR	p_mild_to_moderate	0.19	
From NPDR to severe NPDR	p_moderate_to_severe	0.17	
From severe NPDR to PDR	p_severe_to_PDR	0.29	
From PDR to blindness	p_PDR_to_blindness	0.21	
Health utility value [51]			
NDR	u_NDR	0.94	0.83–1.05
Mild NPDR	u_NPDR	0.87	0.73–1.01
Moderate NPDR	u_NPDR	0.87	0.73–1.01
Severe NPDR	u_NPDR	0.87	0.73–1.01
PDR	u_PDR	0.83	0.74–0.92
Blindness	u_blindness	0.81	0.73–0.89
Natural mortality rate (‰) [48]	pDie	7.07	±25%
Death multiplier [49]			
Diabetes	mortality_multipliers_diabetes	2.34	2.22–2.46
Blindness	mortality_multipliers_blindness	1.9	1.04–2.7
Relative risk of laser treatment [47]	RR_laser	0.49	0.37–0.64
Sensitivity (%) [43,44]			
Ophthalmologist screening	sen_op	96	94.79-97.21
AI screening	sen_AI	90.79	86.40-94.10
Specificity [43,44]			
Ophthalmologist screening	spe_op	94.67	94.57–97.43
AI screening	spe_AI	98.50	97.80–99.00
Discount rate (%) [52]	discount_rate	3	0–6

### Markov queue analysis results

**Fig 3:**
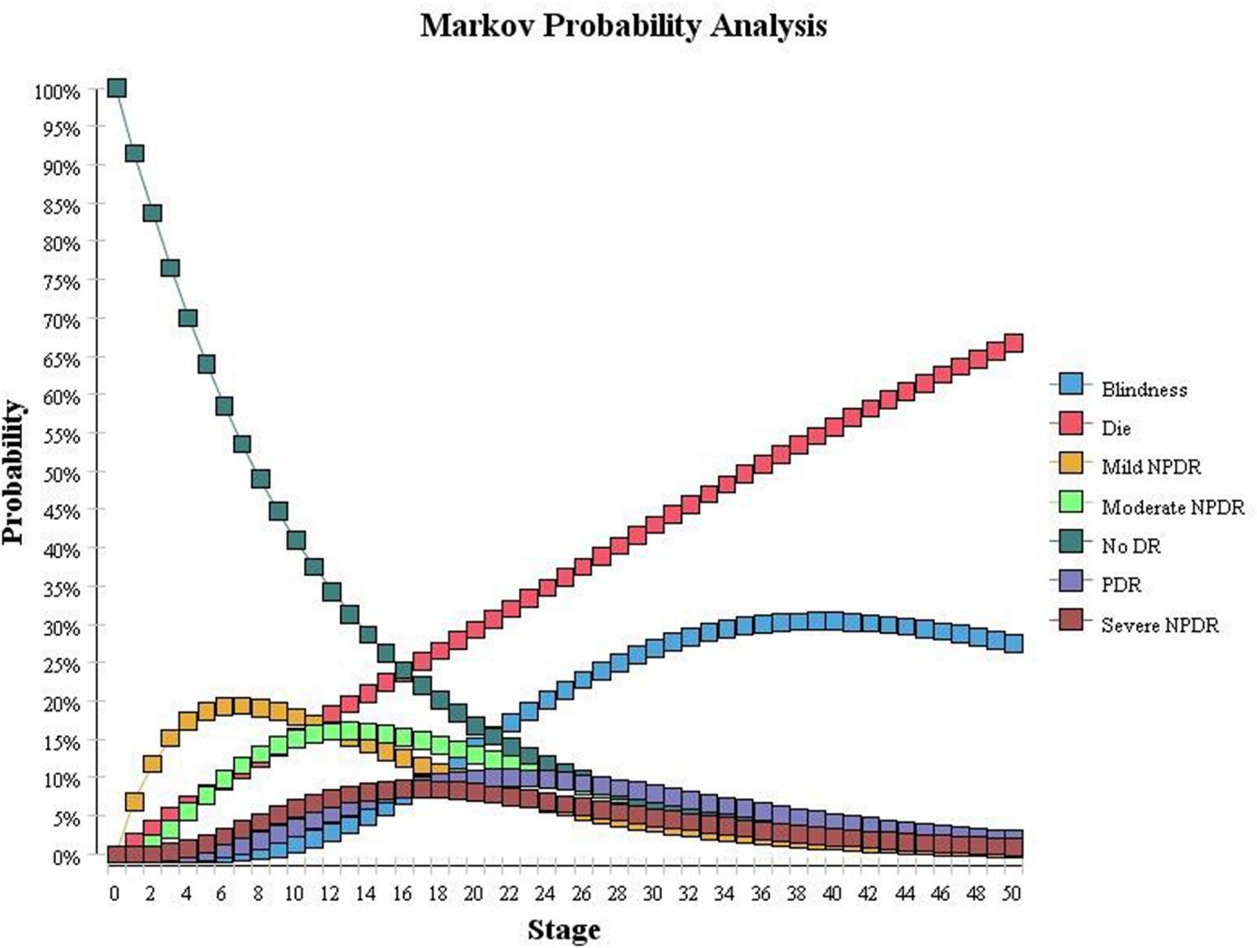
Distribution probability of unscreened Markov queues.

In this study, Markov queue analysis of the Markov model was used to simulate the distribution probabilities of various health statuses of 10,000 diabetic patients in rural areas in the next 50 years without DR screening and any intervention, as shown in Fig 3 (the ordinate is the distribution probability, which is the cycle). In the model, the population with DM at the initial stage was alive without DR. The population with DR showed a natural course of disease and gradually changed to different health states over time. The number of cases of NDR gradually decreased, while the number of cases of DR and death gradually increased.

When the model was run for 5 cycles, 6.5% of the population had died, and 69.57% of the 93.5% diabetic population survived without DR, and 30.43% survived with DR. According to a report from a prospective study in Shanghai [56], people with type 2 diabetes and DR had a 5-year cumulative incidence of 46.89%. Considering that this study assumes that the simulation model runs in each cycle, the survival of individuals with DR occurs only in the original state of development and progresses to the next phase of the new state of the two transition paths. For example, if the individuals with NDR in the model do not develop mild NPDR within 1 year, they can only remain in the NDR state and will not transfer to moderate NPDR or other DR states. Therefore, the incidence of DR among these individuals may be lower than that in real life, resulting in the 5-year cumulative incidence of simulated DR in this study being slightly lower than the results reported in the Shanghai study. Other studies have also reported a 5-year cumulative incidence of DR of 3.9%-47.5% [57–59], which indicates that the simulation results of this study are relatively reasonable.

When the model ran for 50 cycles, 66.6% of the population entered the state of death, while only 3.4% of the 33.4% of the population that survived remained in the state of NDR. The remaining 96.6% of the diabetic population developed DR, among which 2.1% developed mild NPDR, 3.9% developed moderate NPDR, and 3.0% developed severe NPDR. A total of 5.9% developed PDR, and 84.9% developed blindness.

### Cost-effectiveness analysis results

The Markov decision model for DR was used to analyze the cost-effectiveness of each DR screening strategy. The model simulated and estimated the total cost per capita and QALYs of the rural diabetic population under different screening strategies after 50 years. The results are shown in Table 9. From the perspective of the health system, AI-based screening was more costly (incremental cost: 37,257.76 yuan (5,182.25 US dollars)) but more effective (incremental effectiveness: 0.33 yuan (0.05 US dollars)) than no screening. Compared with AI-based screening, ophthalmologist screening was more costly (incremental cost: 14,886.76 yuan (2,070.63 US dollars)) and less effective (incremental utility: -0.31). Compared with no screening, the ICER of AI-based screening was 112146.99 yuan (15,598.72 US dollars)/QALY, which was lower than the ICER threshold (it was lower than 3 times the GDP per capita, which was 217341 yuan (30,230.36 US dollars)). Therefore, AI-based screening is cost-effective, and the increased cost of each additional QALY is merited.

**Table 9:** Cost-effectiveness results from the health system perspectives (the cost was calculated based on a Chinese yuan: US dollar ratio of 7.189: 1).

	Effectiveness (QALYs)	Incremental effectiveness (△QALYs)	Cost (US dollars)	Incremental cost (△US dollars)	ICER (△US dollars /△ QALYs)	
No screening	16.83	0.00	0.00	0.00	0.00	
AI screening	17.17	0.33	5182.25	5182.25	15598.72	
Ophthalmologists screening	16.86	-0.31	7252.87	2070.63	-6708.27	(Dominated)

Notes: QALYs: Quality-adjusted life years; ICER: Cost-effectiveness ratio.

**Fig 4:**
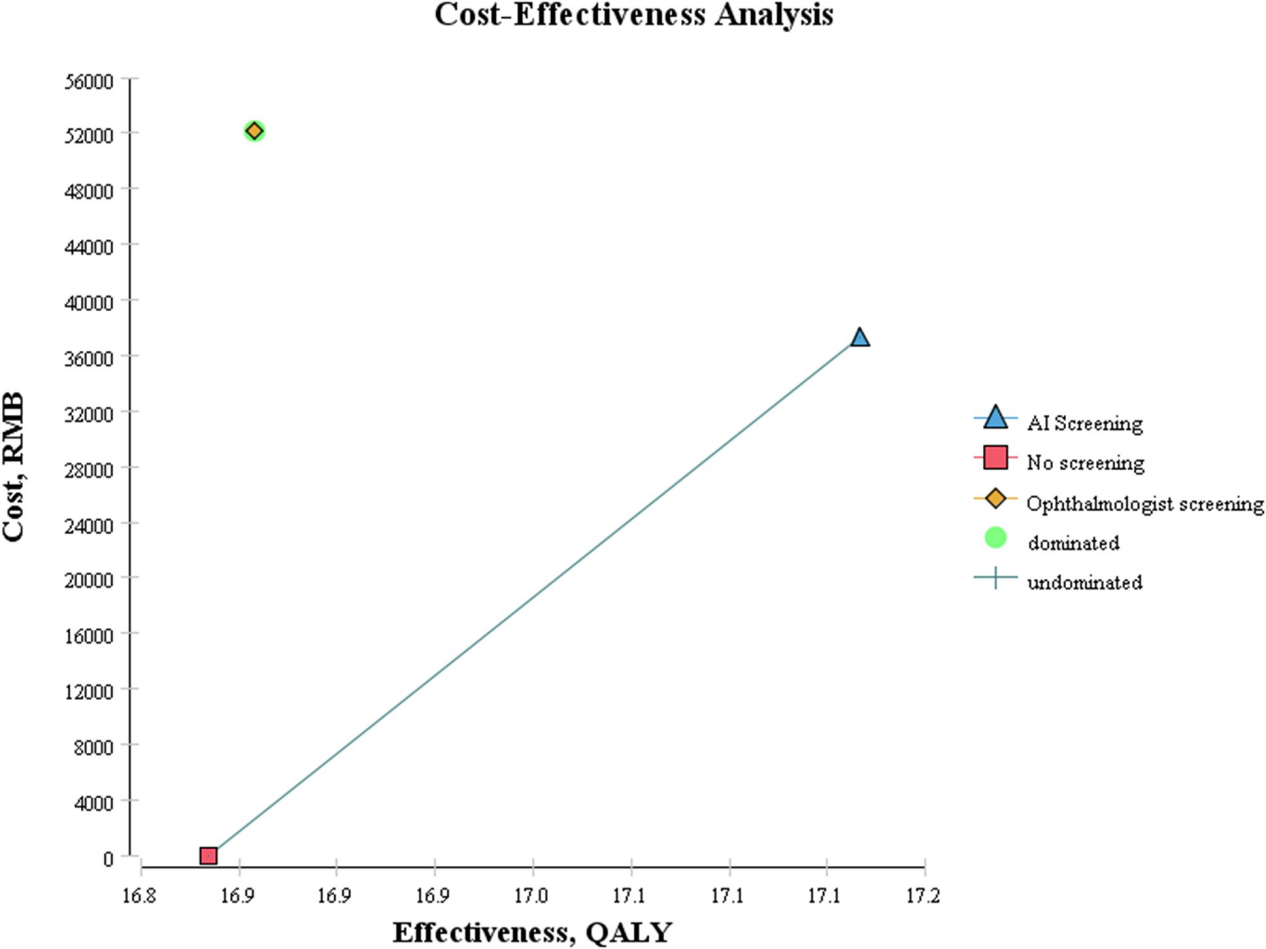
Cost-effectiveness diagram of all DR screening strategies.

The cost-effectiveness results are shown in Fig 4. In the cost-effectiveness diagram, the abscissa represents the effect (QALYs, year), and the ordinate represents the cost (US dollars). The cost effect of all strategies is connected by line segments, which are defined as the cost-effect boundary curve. The slope of a line segment connecting two points is the ICER. The lowest-cost strategy is part of the boundary; if this strategy is superior to alternative strategies, there is no boundary curve, and the no screening strategy is represented by the red square in the lower left corner of the figure.

At the lower right side of the cost-effectiveness boundary curve, the dominant strategy represents the strategy with small cost and large effect. This means that when there are multiple strategies to choose from, the dominant strategy is better than the other strategies. Dominant strategies, in the upper left-hand side of the cost-effectiveness boundary, represent high-cost, low-performance strategies, and they include strictly dominated strategies, in which one strategy out of a group is consistently better than the strictly dominated strategy and is thus eliminated in the analysis. The cost-effectiveness boundary curve is not included. The yellow diamond in the upper right corner represents the ophthalmologist screening strategy, which is the absolutely dominated strategy.

All the strategies on the cost-effectiveness boundary curve are relatively ideal choices. The AI-based screening strategy represented by the blue triangle in the figure needs to be selected by the decision-maker according to whether the ICER of the AI-based screening strategy is lower than the ICER threshold (1–3 times China’s GDP per capita in 2020, 9,990.68–30,230.34 US dollars) to evaluate whether the strategy is cost-effective.

### Results of sensitivity analysis

#### Single-factor sensitivity analysis

**Fig 5:**
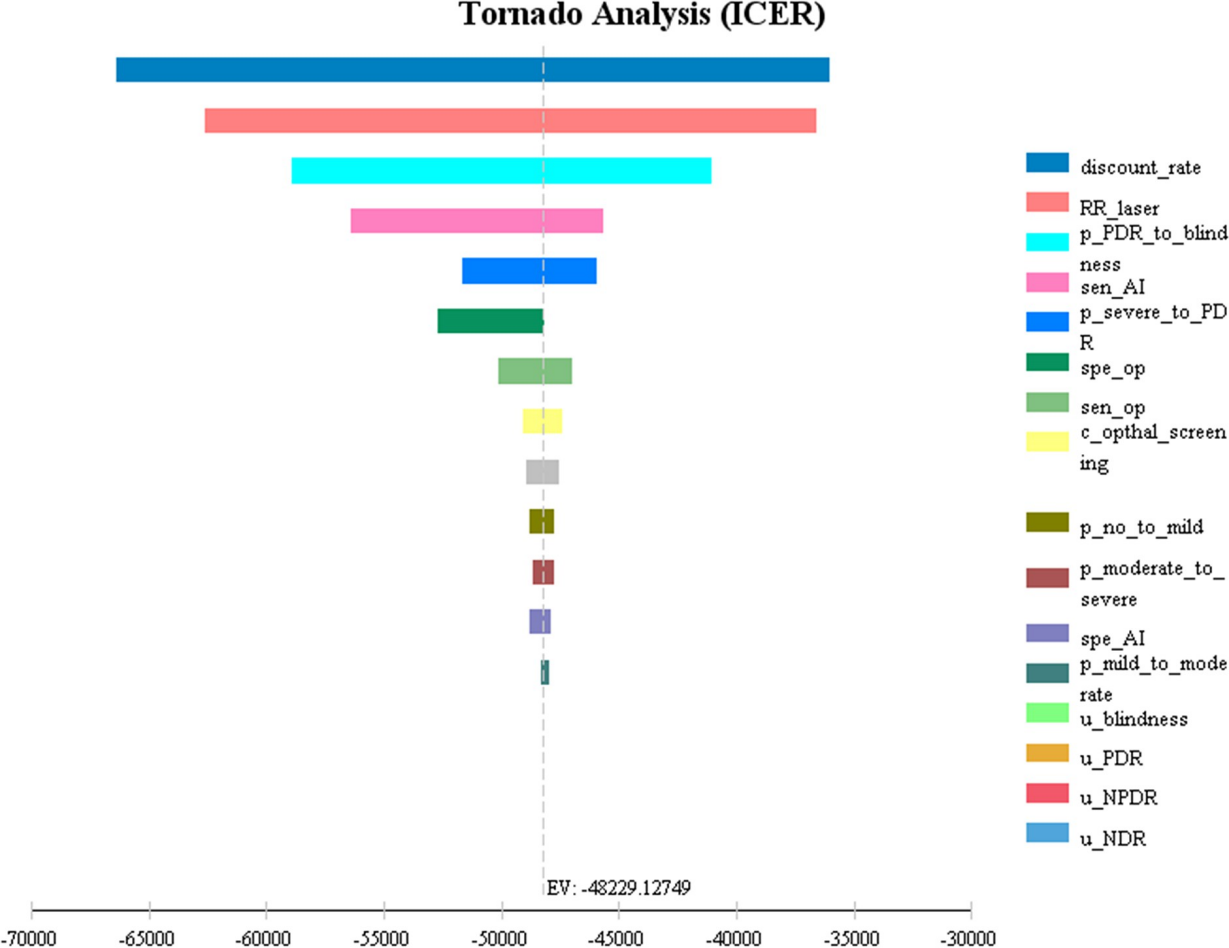
One-way sensitivity analysis (tornado diagram) under the health system perspective. Abbreviations: ICER = incremental cost-effectiveness ratio; RR = relative risk; p = transition probability; sen = sensitivity; spe = specificity; c = cost; op = ophthalmologist screening; AI = AI-based screening; u = utility; NDR = nondiabetic retinopathy; NPDR = nonproliferative diabetic retinopathy; PDR = proliferative diabetic retinopathy.

One-way sensitivity analysis was conducted on the model to observe the impact of model parameters on the results. As shown in Fig 5, compared with ophthalmologist screening, AI-based screening had the most influential three variable parameters from the perspective of the health system: discount rate, the relative risk of laser treatment and the probability of the transition from PDR to blindness, followed by the sensitivity of AI-based screening, whereas the health utility value had little effect on cost-effectiveness.

#### Probability sensitivity analysis

The model was analyzed with probability sensitivity. Monte Carlo simulation technology was used to conduct 10,000 sampling simulations on the model, and the cost-effectiveness of each screening strategy was obtained under a series of different WTP thresholds and changing the values of multiple parameters in the model at the same time. Incremental cost-effectiveness changes and the probability of cost-effectiveness could further determine the stability of the results.

**Fig 6:**
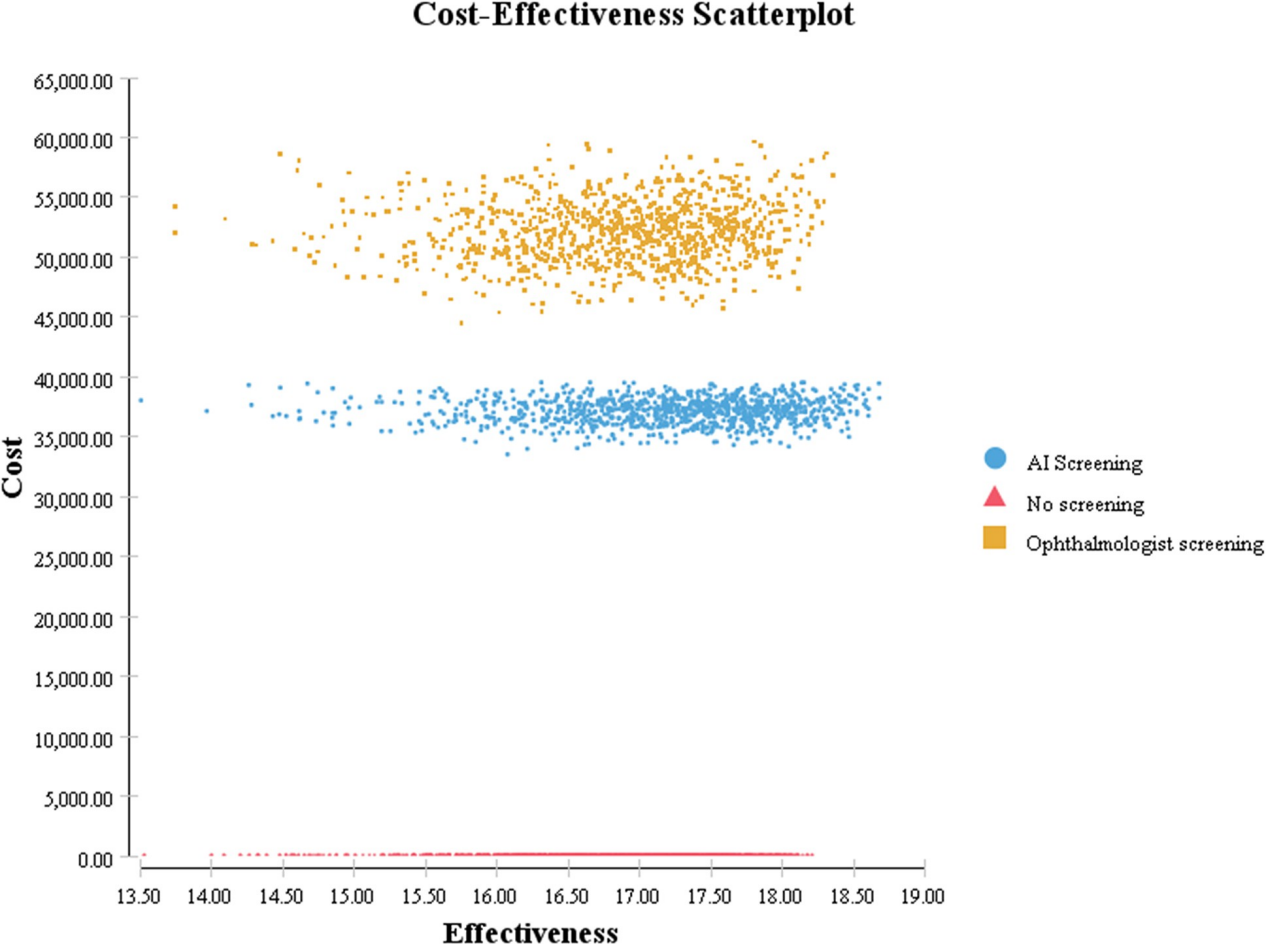
Cost-effectiveness scatter plot.

From the perspective of the health system, Fig 6 shows significant differences in cost and effectiveness distribution among screening strategies, and the AI-based screening strategy is still dominant compared with ophthalmologist screening.

**Fig 7:**
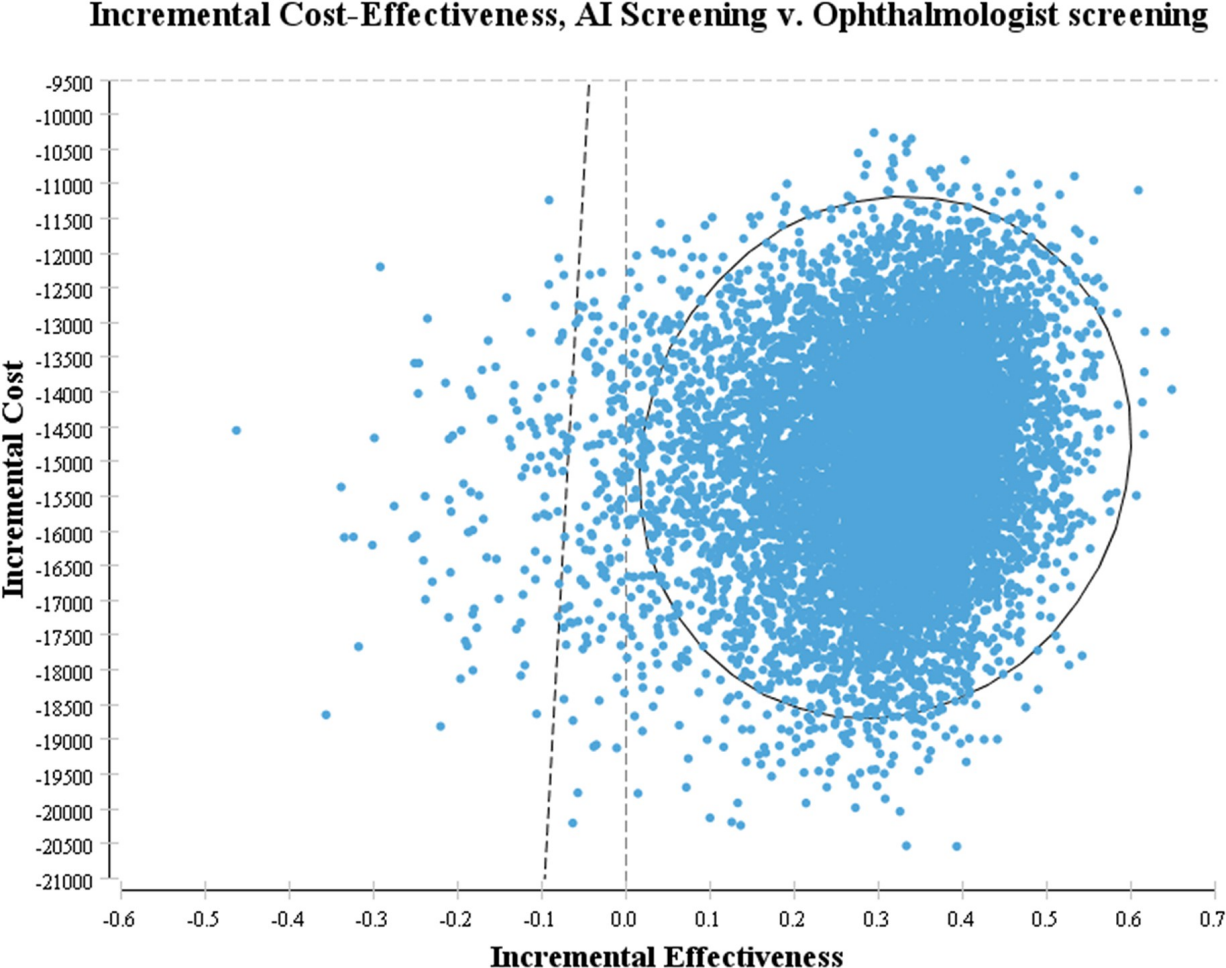
Incremental cost scatter plot.

Fig 7 shows that when compared with ophthalmologist screening, the scatter plot points of AI-based screening are almost all concentrated in the lower right part of WTP, and most of the scatter plot points of incremental cost and incremental effectiveness are located in the fourth quadrant, indicating that the AI-based screening strategy has the absolute advantage.

**Fig 8:**
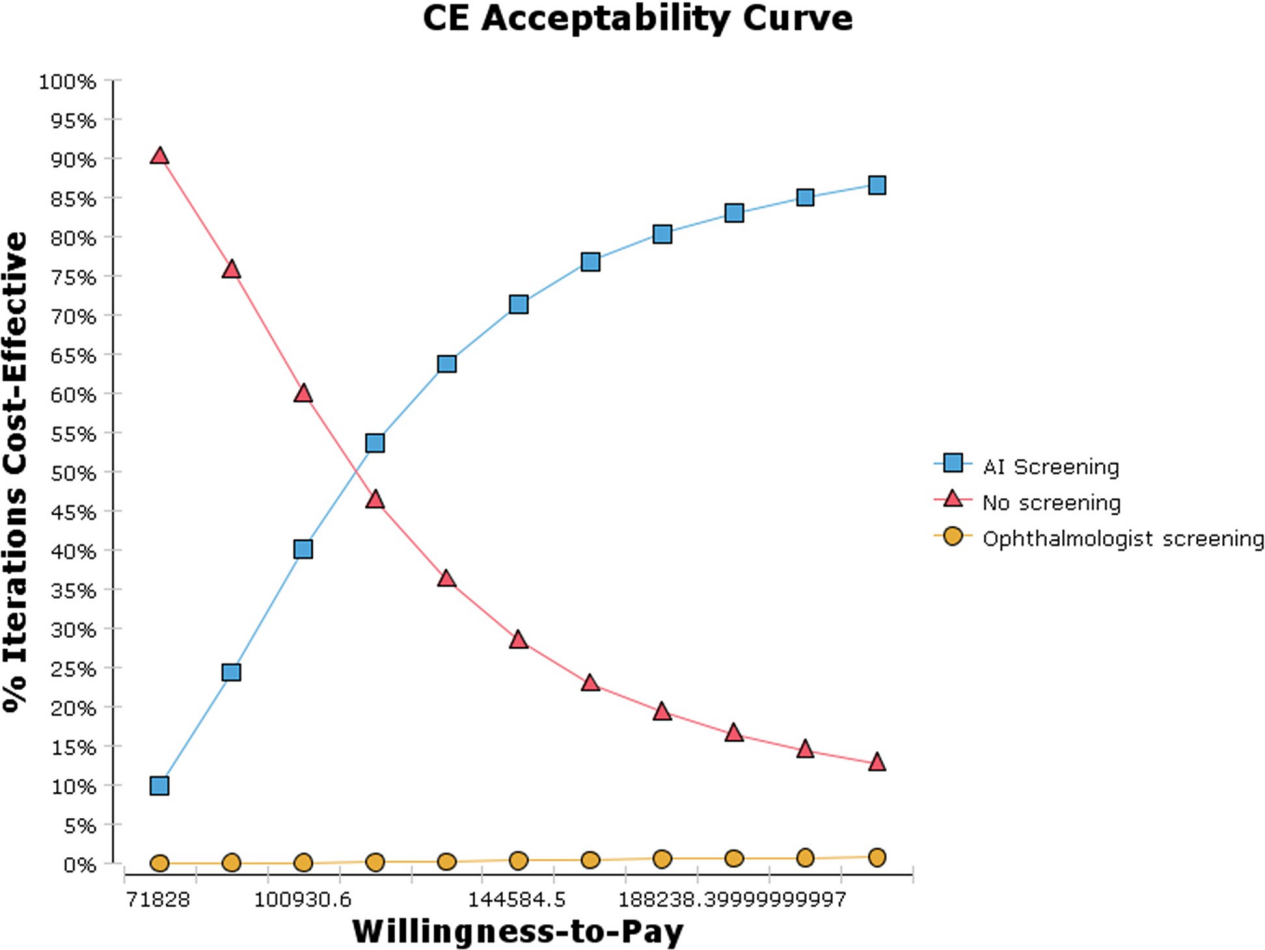
Cost-effectiveness acceptance curve (CEC).

Fig 8 shows the cost-effectiveness acceptance curve, which shows that under any WTP threshold of the model, the probability of the cost-effectiveness of each screening strategy increases with the increase in WTP value; in contrast, the probability of the cost-effectiveness of no screening decreases. At the lowest WTP threshold (1 times the GDP per capita, 71828.0 yuan (9,990.68 US dollars)), the cost-effectiveness probabilities of no screening, AI-based screening, and ophthalmologist screening were 93.4%, 6.6% and 0, respectively. At the maximum WTP threshold (3 times the GDP per capita, 217,341.0 yuan (30,230.34 US dollars)), the probabilities of the cost-effectiveness of no screening, AI-based screening, and ophthalmologist screening were 11.5%, 87.9% and 0.6%, respectively. When the WTP threshold was 115,481.9 yuan (16,062.58 US dollars), AI-based screening was consistently more cost-effective than no screening and ophthalmologist screening.

## Discussion

Due to the shortage of ophthalmologists in Central and Western China (20 per million people), ophthalmologist screening is difficult to carry out continuously in rural areas. In contrast, the DR results of AI-based screening are fast and accurate [16,17], and the cost of per capita annual screening is lower, which was 25.01 yuan (3.48 US dollars) for each person in this study. AI-based screening reduces the transportation costs and labor loss costs of DM patients on their way to hospitals or ophthalmologists in rural areas for screening, saves labor costs and social resources, reduces the occurrence of DR and improves patients’ quality of life, so it is economical and effective to promote in rural areas. In the study by Huang et al., the Markov model was first used to evaluate the health economics of applying AI-based screening for DR in rural areas of China, and AI-based screening was expected to become an alternative method for DR screening in rural areas of China, which is similar to the results of this study [37]. In addition, compared with ophthalmologist screening grading, Xie et al. in Singapore reported that the automatic screening mode based on AI technology saved 14.3% of the cost, but the semiautomatic screening mode combining AI and manual grading was the most cost-effective, saving 19.5% of the cost [60]. In the UK, Scotland et al. found that using AI automatic grading could reduce the manual grading time and labor cost to nearly zero in the screening process [61], and Tufail et al. reported that AI-based screening could save 12% to 21% of the cost [62]. The results of this study show that compared with manual grading, automatic grading of AI screening can save 28.5% of the cost for each diabetes patient. The difference in cost savings between this study and those in Singapore and the United Kingdom may be due to different manual grading screening schemes (this study and the United Kingdom study had three grading stages, while the Singapore study had two grading stages), different research groups (this study had Asian researchers, the United Kingdom study had white researchers, and the Singapore study had Asian and white researchers), different DR classification methods (different classification and referral criteria for diabetic retinopathy), different screening rates, different discount rates (3% in this study, 3.5% in the UK study, and the Singapore study did not use discount rates), different screening costs (this study included treatment costs, but the UK and Singapore studies did not, and the maintenance costs of various AI screening systems and equipment were different). With the continuous progress of AI technology, the performance of AI diagnosis has further improved. Therefore, AI-based screening will effectively relieve the issue of ‘the shortage of ophthalmologists in rural China in the future, reduce the work pressure on ophthalmologists, and reduce the economic pressure on DR patients.

This study based on a Markov model for the cost-effectiveness of DR screening showed that the screening interval was 1 year among people with type 2 diabetes in rural areas. From the perspective of the health system, the lowest-cost strategy was no screening, but the effect was the worst. The ophthalmologist screening effect was better than that of no screening, but it was more expensive, so it was an inferior strategy in this study, which indicates that it was once a dominant strategy but now needs to be eliminated. Compared to the first two strategies, AI-based screening was the dominant and the most cost-effective. From the perspective of the health system, compared with ophthalmologist screening, AI-based screening saved 37257.76 yuan (5182.25 US dollars) for each DM patient and increased quality of life years by 0.33 years. The cost-effectiveness analysis results of AI-based screening, namely, the ICER, were 112146.99 yuan (15,598.72 US dollars)/QALY, less than 3 times China’s GDP per capita in 2020.

Sensitivity analysis was also conducted in this study to evaluate the influence of uncertainties in the model on the ICER of each DR screening strategy and to test the reliability of the cost-effectiveness analysis results. The results of single-factor sensitivity analysis showed that the key factors that affect the cost-effectiveness of DR screening included the discount rate, the relative risk of laser treatment, the probability of the transition from PDR to blindness and the sensitivity of AI-based screening. This was similar to the findings in the study by Ben et al. [35], in which the parameters that had the greatest influence on the ICER were binocular blindness, the utility value of STDR, the discount rate of the relative risk of laser treatment and the cost of STDR health status. In addition to the cost discount rate and the sensitivity and specificity of each screening strategy, the prevalence of adherence to DR screening was also included in the report by Kim et al. [29]. DR screening is more challenging in the case of ophthalmic medical resource shortages in rural China, which has a higher prevalence of DR [2]. By relying on the site survey results of this study, the main influencing factors of participation in paid DR screening included low awareness of DR, neglect and high cost. The results are similar to those reported in a study in Hong Kong [63]. Therefore, before widely carrying out DR screening, in addition to strengthening DR health education for DM patients, reducing the self-paid component of the screening will increase patients’ screening adherence, thus improving the cost-effectiveness of screening. While decision-makers are constrained by budget when choosing screening strategies and investing resources, they need to select the most cost-effective strategy according to the maximum additional cost that decision-makers are willing to pay for the unit effect of screening, namely, the maximum WTP value. The results of the probabilistic sensitivity analysis of this study showed that, based on the WTP threshold range of 1–3 times the GDP per capita, it was not cost-effective to screen when the WTP threshold was 71828.0 yuan (9990.68 US dollars); AI-based screening was always more cost-effective than no screening and ophthalmologist screening when the WTP threshold was 115,481.9 yuan (16,062.58 US dollars). At present, no systematic DR screening program has been established in China, and the cost-effectiveness results of different DR screening strategies vary depending on the research purpose, the research perspective and regional background, so the results of this study are intended to provide theoretical support for the development of DR screening programs in the future.

This study has some limitations. First, to simplify the calculation of the model, it was assumed that the adherence of DM patients in the AI-based screening and ophthalmologist screening groups to participate in DR screening, follow-up examinations and treatment was 100% every year. In fact, only 58%-81% of DM patients could be regularly screened [63], so the estimated cost effect of the model would be lower than the actual cost effect. Second, the sensitivity and specificity of AI-based screening and ophthalmologist screening in this project could not be obtained because the DR diagnosis results of those who were screened for positive DR and received fundus fluorescein angiography, which was the gold standard examination, were not followed up in this study; considering that the AI diagnosis system is quite mature at present, the sensitivity and specificity were 97% and 87% [64], respectively. Therefore, the sensitivity and specificity of AI-based screening in the study by He et al. [43] and DR screening by an ophthalmologist using a fundus camera in the study by Li et al. [44] were adopted in the model, resulting in the cost-effectiveness of the two screening strategies possibly differing from the actual situation. Third, this study assumed that the DR incidence, conversion probability, and mortality rate in the Markov model remained unchanged throughout the model cycles. Moreover, the time changes in the simulated parameters were not stratified by age, but the corresponding values actually increased with increasing age in the process of DR development and prognosis. Therefore, the final cost effect of the model simulation would be lower than the actual cost. Fourth, this study assumed that individuals in the model could only transition to one state according to the set transition path within each cycle but could not transition to multiple states at the same time. In addition, DR patients were not allowed to recover to a complete health state, that is, a no DR state. Therefore, in the cohort analysis of the Markov model, the simulated distribution probability of each DR state after 50 years and the cost-effectiveness of each screening strategy will be lower than the actual distribution probability. Fifth, the treatment cost of DR patients in this study has not been collected in detail, so the estimated cost is lower than the actual cost. Sixth, this study did not analyze the data of patients with diabetic macular edema and DR, so the impact of these data affecting the overall cost-effectiveness was ignored.

AI-based screening not only saves money but also improves the overall quality of life of DM patients and is the most cost-effective when compared to no screening and ophthalmologist screening for DR. Therefore, it is economically effective and feasible to promote AI-based screening in rural areas of China and other regions with similar economic and social characteristics worldwide. The results of this study can provide a scientific basis for the further development of early screening programs for DR.

## Supporting information

S1 Fig Markov decision model of the nonscreening group. (JPG)

S2 Fig Markov decision model according to ophthalmologist screening. (JPG)

S3 Fig Markov decision model according to AI screening. (JPG)
